# Hirudotherapy for limb ischemia in the pediatric intensive care unit: A retrospective observational cohort

**DOI:** 10.3389/fped.2022.1011171

**Published:** 2023-01-05

**Authors:** Joseph C. Resch, Rachel Hedstrom, Marie E. Steiner, Sameh M. Said, Arif Somani

**Affiliations:** ^1^ Department of Pediatric Critical Care, University of Minnesota, M Health Fairview Masonic Children’s Hospital, Minneapolis, MN, United States; ^2^ Department of Pediatric Cardiac Surgery, University of Minnesota Masonic Children’s Hospital, Minneapolis, MN, United States

**Keywords:** hirudotherapy, leech, limb ischemia, symmetric peripheral gangrene, ECMO, pediatric, case series

## Abstract

**Background:**

Acute limb ischemia due to microvascular malperfusion may be refractory to initial therapies. Medicinal leech therapy (hirudotherapy) has been attempted in plastic and reconstructive surgery to improve venous congestion in ischemic flaps; however, there are minimal reports related to ischemia secondary to arterial malperfusion. We evaluated a pediatric cohort from an academic intensive care unit with refractory limb ischemia in whom hirudotherapy was attempted to elucidate its use and outcomes.

**Method:**

Institutional patient database was queried to identify pediatric patients (<18 years) who received hirudotherapy in the pediatric critical care unit and met inclusion/exclusion criteria. Patient charts were evaluated for indices including demographics, primary disease, coagulative status, vascular access, vasoactive medication dosing, bleeding, leech use, limb and mortality outcomes. Data was evaluated to identify trends or suspected impact on outcomes.

**Results:**

Hirudotherapy was used in 7 patients for limb ischemia, 5 with congenital heart disease, and 2 others with viremic shock. Time to leech application following recognition of ischemia averaged 3 days, with duration of use averaging 3.9 days. Five patients discontinued therapy due to bleeding. Mortality rate was 57%, all secondary to multiorgan failure. In 3 surviving patients, 4 of 5 treated limbs resulted in at minimum partial amputation. Vasoactive-inotropic score tended higher prior to leech application, suggesting a vasoconstrictive pathway for arterial malperfusion. No identifiable trends appeared associated with salvaged limb or adverse effects. Blood loss predictably increased with leech application, as did total transfusion requirement.

**Conclusion:**

This case series establishes baseline data for use of hirudotherapy in critically ill children with acute limb ischemia caused by arterial malperfusion. Based on this retrospective cohort, we cannot recommend routine use of hirudotherapy for acute limb ischemia from arterial malperfusion in the pediatric intensive care unit. Application of leeches should be aligned with a protocol defining start and stop parameters, standardized leech utilization, and monitoring for adverse outcomes. Future study would benefit from consensus definitions of study outcomes, including perfusion recovery, tissue/limb salvage and bleeding manifestations. Additional prospective studies are needed prior to any standard or systematic recommendations for use.

## Introduction

Humans have leveraged the enzymatic properties of leeches since the earliest documentation of medicine. Hirudotherapy, as is known, has been utilized for pathologies ranging from hysteria to hemorrhoids (1, 2), but is currently only approved by the U.S. Food & Drug Administration for improving venous congestion in graft tissue (3, 4). Reconstructive surgery has established extensive systematic exploration of hirudotherapy in digit and flap reconstruction for this purpose, with variable guidance on leech utilization (5–11). Literature in hirudotherapy is expanding exponentially, reflecting increased interest in a variety of applications (12).

Acute limb ischemia is a manifestation of poor perfusion that is associated with high morbidity and mortality rates which may be refractory to medicinal or endovascular therapies. Thrombotic etiologies predominate in pediatrics, primarily catheter-associated in infancy and trauma in children and adolescents (13). This contrasts with adults, where the majority of cases are secondary to peripheral artery disease (14). Within the context of critical disease, ischemia may be encountered simultaneously in multiple limbs, with proposed contributions from sepsis, disseminated intravascular coagulopathy (DIC), low blood flow states, and vasopressor use (15–19). Diffuse or bilateral ischemia isolated to acral limbs is termed symmetric peripheral gangrene (SPG), and may be difficult to treat without surgical intervention (16, 19).

The primary mechanism of action for hirudotherapy in surgical fields is bloodletting for venous congestion, which may reduce interstitial pressure and improve arterial inflow. Additionally, leech saliva possesses anticoagulative and platelet inhibition properties (9, 20, 21). Bivalirudin, commonly used as a second line systemic anticoagulant, is a recombinant form of the leech enzyme hirudin (22). This lends biologic plausibility for its use in local malperfusion due to arterial insufficiency in pediatric patients when standard therapy fails. Adult patients with peripheral artery disease appear to be the most comparable population for this use, with mixed results (23, 24). To our knowledge, this approach has only been reported in two pediatric cases (25, 26). The pediatric critical care team at our institution has attempted to exploit this quality for treatment of refractory peripheral limb ischemia in select cases. No established guidance for this use exists, and the same salivary enzymes that dictate leech's therapeutic potential also predispose to adverse effects (2, 27–33). We have previously reported a neonate requiring extracorporeal membrane oxygenation (ECMO) on whom hirudotherapy was attempted for limb ischemia, resulting in massive hemorrhage (34).

This paper aims to expand available data for hirudotherapy in acute limb ischemia with a retrospective case review, attempting to identify any predictable trends to help guide its practice or restrict its use. We hypothesized that bleeding complications may counter potential benefits in outcome for critically ill children.

## Methods

This study was approved by the University of Minnesota institutional review board associated with MHealth Fairview Masonic Children's Hospital. We performed a literature search of both Medline and PubMed using combinations of the search terms “hirudotherapy”, “leech therapy”, “children”, “ischemia”, “arterial ischemia” and “limb ischemia” to assess the use of leeches for our indication. The virtual pediatric systems (VPS) database for Fairview Health System at our institution was polled for all patients who received medicinal leech use in the Pediatric Intensive Care Unit (PICU). All patients were screened for inclusion and exclusion criteria. Those whose charts did not indicate that they had consented to their medical records being queried for research were excluded. Patients were included if chart review indicated arterial malperfusion and excluded if leeches were used for venous congestion (specifically in reconstructive surgery). Nine patients were identified between 2012 and 2020, two which were excluded due to treatment indications of venous congestion in flap repairs. Data variables were selected to evaluate contributors to ischemia and coagulopathy, leech use, bleeding, and outcomes, all which were compiled in REDCap patient-secure database. Vasoactive-inotropic scores (VIS) were quantified using the formula proposed by Koponen et al. (35). Data was divided into a pre-leech phase (3 days), leech phase (duration of hirudotherapy plus 1 day), and post-leech phase (2 days). The leech phase extended one day beyond leech removal to account for additional bleeding based on our experience with additional side effects and other available literature (6, 34). The best practices integrated informatics core assisted with data extraction from the electronic health record to improve efficiency. More subjective medical information was retrieved by trained research staff. Data was examined to identify any guidance on future decision-making and development of internal protocol. No evaluation for statistical significance was performed given overall paucity of patients and limited literature to support power analysis.

## Results

### Demographics & disease process

Patient demographics are summarized in Table 1. Ages ranged from newborn to 17 years, with slight male-predominance (4/7). Two patients (1, 2) presented for viremic shock, one from H1N1, and another from varicella who was immunodeficient secondary to bone marrow transplant. The remaining 5 patients (3–7) had congenital heart lesions with ischemia developing within 48 h of procedural repair, transplant, or cardiac arrest. All cardiac patients required ECMO during hospitalization, including at hirudotherapy initiation. Patient 6 had ECMO discontinued on day 2 of leech phase and patient 5 did not have any ECMO in pre-leech phase. All patients demonstrated evidence of hepatic injury (elevated AST/ALT), coagulopathy (abnormal INR ± fibrinogen, PT, PTT), and thrombocytopenia (Supplementary Figures S1, S2). There was no explicit diagnosis of thrombocytopenia-associated multiorgan failure (TAMOF) or ADAMTS13 levels checked. Four patients received antithrombin III repletion. Six patients were either blood type O+ or O−.

**Table 1:** Demographics.

Patient	Age (mo)	Weight (kg)	Sex	Primary disease process	ICU days	Ventilator hours
1	34	13.9	F	Septic Shock (influenza), Coagulopathy	16	120
2	215	93.5	M	Septic Shock (Varicella), ALF, AKI, hx BMT	21	463
3	0.75	3	F	CHD, BT-shunt occlusion, cardiac arrest, Factor V Leiden	24	500+
4	0	4.5	F	CHD, s/*p* cardiac transplant	252	500+
5	64	18	M	CHD, s/*p* Fontan	28	500+
6	143	53	M	CHD, aortic thrombus, cardiac arrest, hx Fontan	278	500+
7	0	2.73	M	CHD, recent septic shock, s/*p* VSD & aortic repair, ALF	32	500+

mo, month; kg, kilogram; ALF, acute liver failure; AKI, acute kidney injury; BMT, bone marrow transplant; CHD, congenital heart disease; BT, blalock-taussig; VSD, ventricular septal defect.

### Ischemia

**Figure 1:**
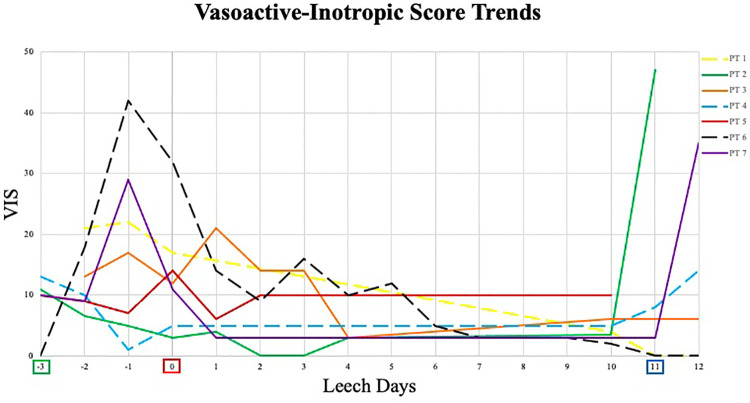
Vasoactive-inotropic score trends. 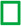 Begin Pre-Leech Phase. 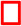 begin leech phase. 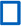 begin post-leech phase. VIS, vasoactive-inotropic score; PT, patient; - - -, surviving patient; —, deceased patient. Of note, leech phase duration was not uniform in all patients; lines were made continuous for clarity of trends – see Table 3 for individual leech application.

Ischemic details are summarized in Table 2. Six patients had bilateral ischemia, one including involvement of 3 limbs. The lone patient with single limb involvement (R foot and toes) had no ultrasound evidence of thrombosis. She did, however, have heart catheterization through R femoral vessels ∼6 weeks prior to ischemia, lending credibility to iatrogenic etiology. All patients required vasopressor support in pre-leech phase (Figure 1) with at least one day reaching VIS 10. Vasopressor use was the primary etiology suggested for ischemia on reviewed progress notes. Time from initiation of vasopressors to recognition of ischemia averaged 3.3 days (range 0–7). Thrombosis was visualized on ultrasound in 3 patients, two with arterial involvement. One of those had single-vessel arterial thrombosis, which could not account for bilateral ischemia. Patients with cardiac procedures all had interventions (heart catheterization, ECMO cannulation, or arterial line) which correlated with at least 1 of their affected limbs, and all had a major procedure within 30 days, supporting potential contribution of iatrogenic vessel injury and resultant ischemia. Femoral cannulation for ECMO was identified in only one patient, the remaining either accessed centrally or *via* neck vessels. Four patients received continuous renal replacement therapy. No patients received intravenous immunoglobulin or plasmapheresis. Pre-leech ischemic treatment was not uniform in all patients, with mixed utilization of heparin (5), topical nitroglycerin (6), antithrombin III concentrate (4) and papaverine (7). Aspirin, bivalirudin, endovascular or surgical intervention were not used. There was no ischemic classification (e.g., Rutherford) documented.

**Table 2:** Ischemia.

Patient	Location	Vascular	Thrombosis	Proximal procedure (30 d)	Other detail
1	RH, LH, L toes	PICC LUE	Extensive in ¾ extremities (a/v)	N	Vessels too small for cath-directed thrombolysis
2	RF > LF	N	N	N	
3	RLE > LLE	R femoral A-line & cath; L CVL; ECMO cannulation R neck	Diffuse RLE a/v	ECMO cannulation, BT dilation/stent, heart cath	No doppler pulse in RLE
4	RF, R toes	R fem A-line, R fem cath; central ECMO	N (decreased flow)	Heart transplant, ECMO cannulation	Ischemia worse after 5 days (ECMO change)
5	R toes > L toes, RF	R fem cath, L fem A/V; ECMO Avalon	N	Heart transplant, heart cath, PA stent	Worsening near death (leeches on)
6	R toes > L toes, RF	ECMO cannulation R fem A/V, R fem cath	N (decreased flow)	Heart transplant	Extension of ischemia to heel after 24-48 h
7	RF > LF, R toes > L toes	R fem A; central ECMO	R Ext Iliac V occlusion	VSD and Ao arch repair	

d, day; hrs, hours; RH,LH, right, left hand; RF,LF, right, left foot; RLE,LLE, right, left lower extremity; A, artery; V, vein; Fem, femoral; Ext, external; Ao, aortic; N, not present; PICC, peripherally-inserted central catheter; Cath, heart catheterization; CVL, central venous line; ECMO, extracorporeal membrane oxygenation.

### Leech use

Leech application is summarized in Table 3. Leeches at our institution were obtained from Leeches U.S.A., Ltd. There was no institutional protocol for specific indication, application, or monitoring. Hirudotherapy was initiated for severe ischemia refractory to initial topical and/or systemic therapy. The multiorgan disease complexity and general lack of arterial thrombosis presumably inhibited vascular procedural intervention, however this was specifically stated in only one patient's record. The details of hirudotherapy orders were highly variable over the 8-year period as well, ranging from every 1-hour to every 12-hours.

**Table 3:** Leech use.

Patient	Location	Ischemia to leech (d)	Duration of use (d)	Average leeches/day	Max leeches/day	Indication to D/C
1	LH, RH, LF	1	1	5	5	Bleeding
2	RF, LF	5	5	7.8	10	Exam Improved
3	RL	2	5	1.6	3	Bleeding, no effect
4	RF	6	1	1	1	Bleeding
5	RF, R toes	2	3	2	3	death
6	RF, R toes	1	10	3.83	6	Bleeding
7	RF, R toes	4	2	3	4	Bleeding

d, day; D/C, discontinuation; RH,LH, right, left hand; RF,LF, right, left foot; RL, right leg.

Despite bilateral ischemia in six patients, five received leech therapy to only one limb. Time from recognition of ischemia to leech initiation averaged three days (range 1–6). There was high variability in the leech-phase duration, with a maximum of 10 days (although leeches were held for platelet count <50,000/μl in four days). One patient reached completion of intended therapy and one patient died. In all others (5/7) therapy was discontinued secondary to bleeding. Similar high variability existed for number of leeches applied per day (range 1–10) with some correlation to patient size. One patient received only a single leech prior to discontinuation for bleeding. Adequate records were not available for duration between individual leech applications, actual duration of leech feedings, and specific thresholds prior to discontinuation. Records suggested leech attachment for longer than 30–90 min, a behavior which is different than the typical duration of reported leech feeding prior to their detachment (6, 36).

### Leech adverse effects

**Figure 2:**
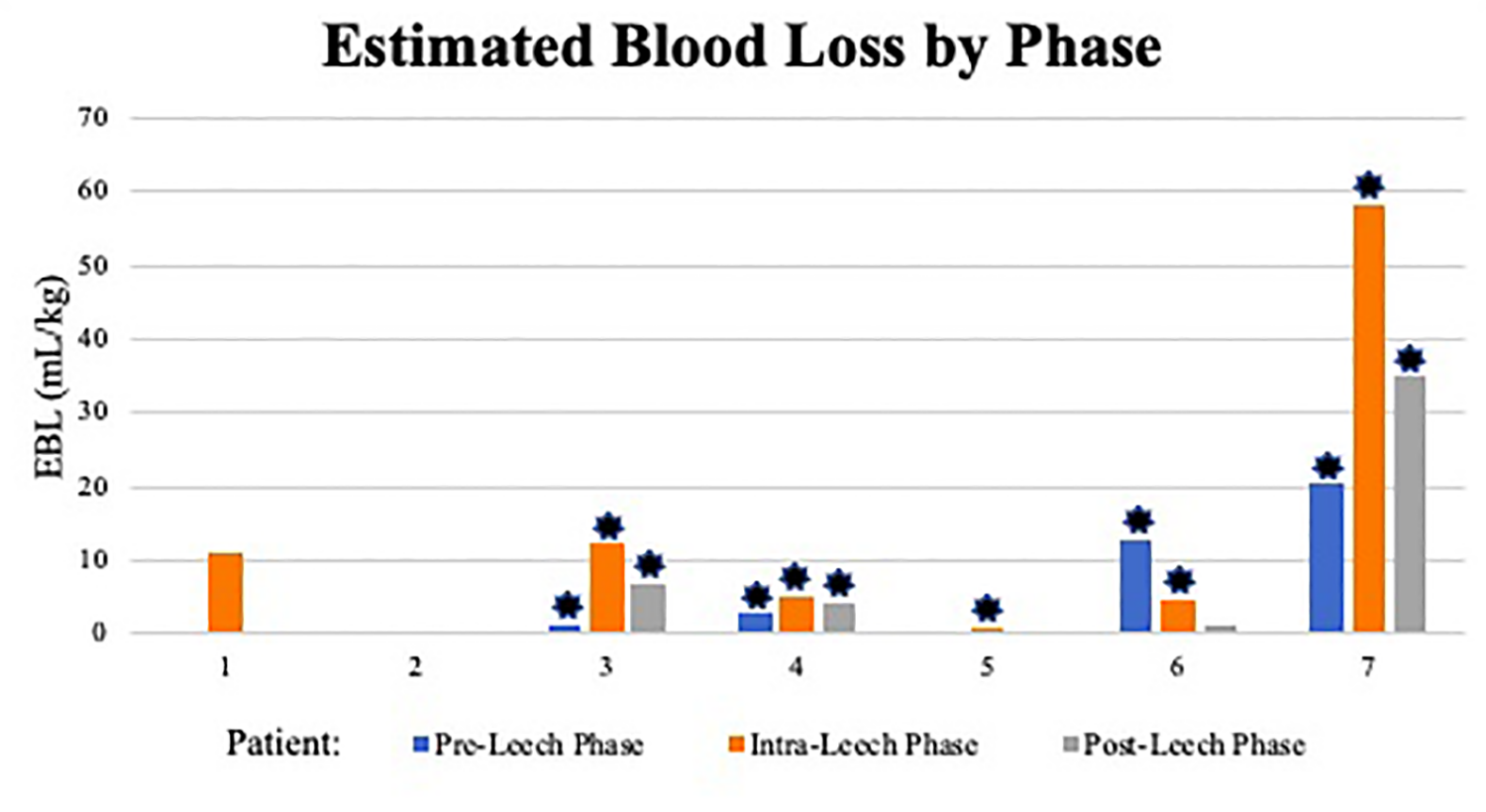
Estimated blood loss by phase. ml, milliliter; kg, kilogram; *: indicates ECMO use within phase.

**Figure 3:**
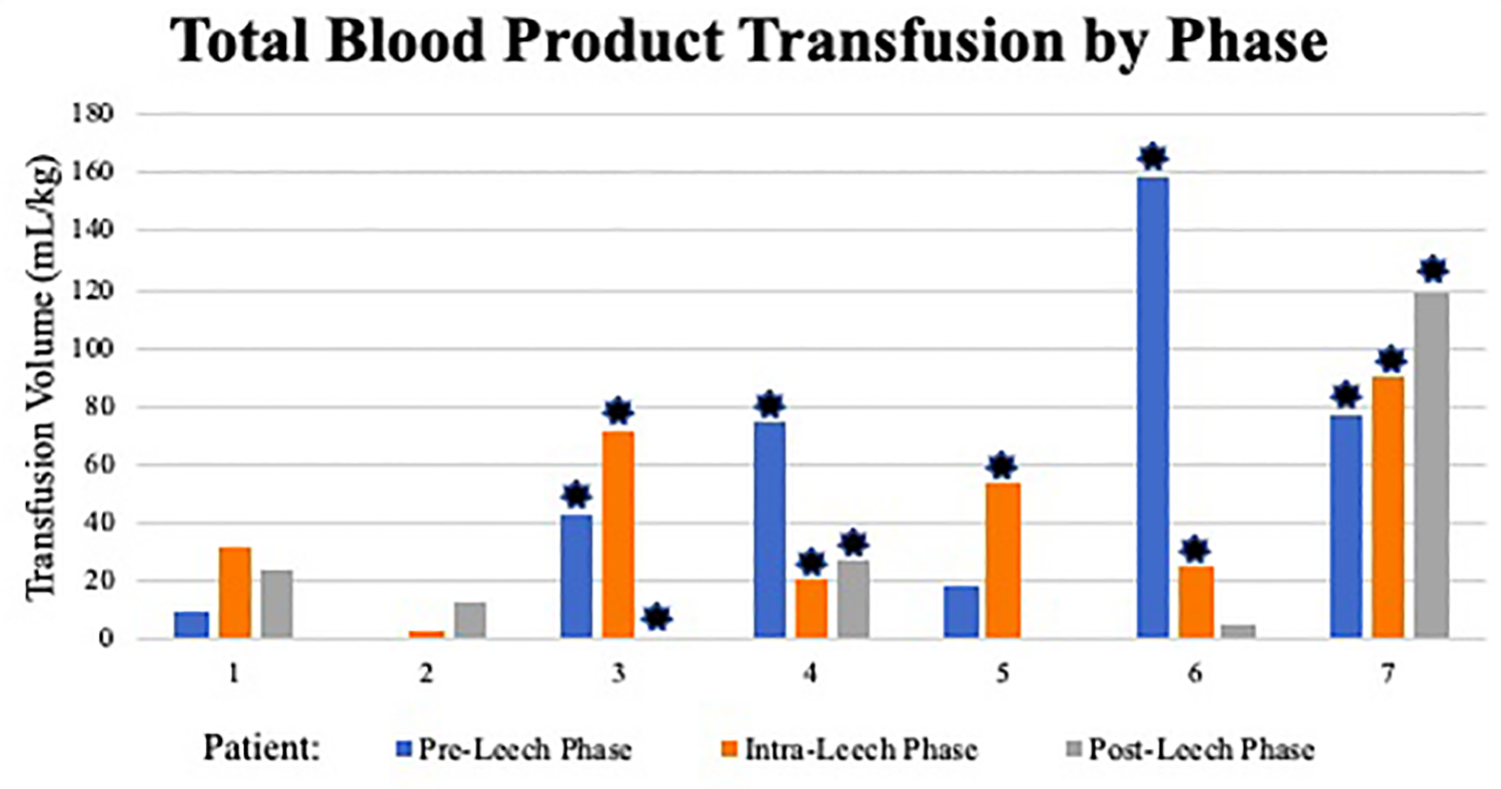
Total blood product transfusion by phase. ml, milliliter; kg, kilogram; *: indicates ECMO use within phase.

There was one positive microbial blood culture following leech therapy within the 2-week post-leech surveillance (*Rhizopus delemar*, patient 7). Other changes to antibiotic regimens were attributed to endotracheal or wound culture positivity, clinically presumed sepsis, or narrowing of therapy. Antibacterial regimens on day of leech initiation included ceftriaxone, cefepime, and meropenem, though appeared directed at other active problems rather than leech prophylaxis. One patient did not have an antibacterial added until day two of leech therapy. There were no allergic or skin reactions identified. Aside from one patient, cutaneous blood loss inherently increased during the leech phase (including 24 h after discontinuation) and decreased in post-leech phase, though with different magnitudes (Figure 2, Table 4). Post-leech phase bleeding appeared to persist to varying degrees. Patient 6 had severe massive hemorrhage in pre-leech phase attributed to ECMO, which outweighed bleeding in the leech phase. Total volume of blood transfusion required increased during leech phase with high variability (1.17–8.17 magnitude increase) and did not consistently downtrend in post-leech phase (Figure 3). Four patients required topical thrombin to bleeding site, with other interventions including pressure dressing, tamponade suturing, and attenuating anticoagulative infusions. Two patients (6 & 7) had severe bleeding specifically at the webbing of great toe. There were no appreciable trends in blood components (hemoglobin, platelet) or coagulative markers (INR), but all values were maintainable at acceptable levels throughout leech phase (Supplementary Figures S1–S3).

**Table 4:** Bleeding indices.

PATIENT	ΔEBL PRE- to LEECH PHASE (magnitude)	ΔTBP PRE- to LEECH PHASE (magnitude)	BLEEDING: DAY LEECH D/C (EBL/kg)	BLEEDING: POST-LEECH DAY 1 (EBL/kg)
1	29	3.25	0.85	21.3
2	Minimal	8.17	0	0
3	10	1.65	28.4	12.3
4	1.72	0.28*	3.33	6.78
5	9.5	2.92	0.69	0.44
6	0.37*	0.16*	0.26	18.1
7	2.8	1.17	58.6	108.4

ΔEBL, change in estimated blood loss; ΔTBP, change in total blood product transfusion; kg, kilogram; *, indicated decrease from pre-leech to leech phase.

### Outcomes

Patient outcomes are summarized in Table 5. Four patients died from multiorgan failure prior to adequate evaluation of limb outcomes. Four of the cardiac patients suffered intracranial stroke (2 ischemic, 2 hemorrhagic), none with identifiable correlation to hirudotherapy or resultant medication changes. Of the five affected limbs treated with leeches in surviving patients, only one limb has remained free of procedural or autoamputation (L foot of patient 1). No identifiable difference in leech use appeared to correlate with limb outcome; in fact, the fully salvaged limb received only one day of therapy (likely 1–2 leeches). Progress notes indicated some element of subjective color improvement in all patients.

**Table 5:** Outcome data.

Patient	Prism3 (death probability %)	28-day limb & mortality	90-day limb & mortality	Overall limb outcome	Cause of death
1	13 (6.26)	Alive; limbs intact	live; autoamputation, amputation	L & R 5th digit autoamputation, amputation L hand; L foot intact	n/a
2	7 (1.08)	Deceased	Deceased	Subjective improvement	MOF
3	35 (98.47)	Deceased	Deceased	No significant improvement	Culture-negative sepsis, MOF
4	3 (1.05)	Alive; limb intact	Alive; limb intact	R great toe amputation, autoamputation remaining R toes, heel improved	n/a
5	3 (0.24)	Deceased	Deceased	Worsening RF & R toes ischemia	MOF, fungal sepsis, ICH
6	6 (0.75)	Alive; limb intact	Alive; limb intact	R great toe & 3rd toe autoamputation; L limb intact	n/a
7	0 (0.5)	Deceased	Deceased	Color improvement, no necrosis improvement	MOF

RF, right foot; PRISM3, pediatric risk of mortality III score (at admission); MOF, multiorgan failure; ICH, intracranial hemorrhage.

## Discussion

We previously reported a neonate requiring ECMO who developed severe hemorrhage with hirudotherapy for limb ischemia (patient 7) (34). This current paper reviews a series of seven critically ill children with acute limb ischemia in whom medicinal leech therapy was attempted for limb salvage. Leech quantity, duration, and decisions for initiation and termination were variable, owing to patient complexity, slow patient accrual over a long study period (2012–2020), and a lack of a consistent protocol. Patients tended to have high VIS prior to ischemic recognition, increased bleeding and blood transfusion requirement during leech therapy, and persistent bleeding beyond 24 h of discontinuation. Mortality was 57% and overall limb salvage was 33% (2/6). Of five limbs treated with hirudotherapy in the three survivors, only one remained free of auto- or surgical amputation at available 2-year follow-up visit.

It is difficult to assess leech efficacy given the patient complexity, multifactorial or unclear etiology for limb ischemia, and non-uniform use of leeches over the study period. Major artery occlusion was only identified in two patients and would not account for witnessed bilateral ischemia in one of them. All five cardiac patients had potential iatrogenic injury from vascular catheters; but this would not consistently account for bilateral ischemia. Low flow state, selective perfusion of deep femoral artery, arterial compression by venous cannulas and high vasopressor requirement are proposed mechanisms of ischemia with ECMO, some which may have additionally contributed in our cohort (37). All but one patient had evidence of bilateral acral ischemia consistent with a diagnosis of SPG. This acral pattern is primarily demonstrated in sepsis or cardiac disorders (17). Recent literature suggests a clinical triad which predisposes patients to SPG: circulatory shock, DIC, and natural anticoagulation depletion often seen after shocked liver (17). All patients in our cohort possessed evidence of hemodynamic insufficiency and consumptive coagulopathy, six with some element of low or falling fibrinogen in pre-leech phase, and five with suspicion or confirmed anticoagulative depletion (low antithrombin III level or received replacement). All patients also had antibiotic use prior to time of leech initiation, suggesting systemic infection and/or inflammation. There is current skepticism toward vasopressor role in SPG. Studies cite a lack of correlation between the initiation of vasopressors and the delayed (often days) clinical presentation of distal ischemia (17, 18, 20). A systematic review for high-dose vasopressor use in septic shock revealed an incidence of acute limb ischemia in 1.6–5.7% of patients (18). Conversely, support for vasopressor-mediated ischemia exists, with vasopressin suggested to be more associated with digital ischemia than other pressors (19, 38). None of these studies evaluated concomitant DIC, coagulopathy, or hepatic dysfunction. Four patients in our cohort developed ischemia between 3 and 7 days after vasopressor initiation while the other three were rapid (within 24 h), so would therefore be difficult to refute potential vasopressor contribution.

Further complicating leech assessment was the overall death rate in an already small cohort (4/7). Acute limb ischemia has high morbidity and mortality in adults, with fatality in 15%–33% and major amputation rates ∼10%–30% (15, 16, 39, 40). The addition of COVID-19 infection or ECMO requirement may increase mortality to 30%–60% (37, 41). On the other hand, physiologic differences may favor pediatric outcomes, including early angiogenesis for collateral vessels, less atherosclerotic presence, and superior vasodilatory response to ischemia (13). Acute limb ischemia in the pediatric population demonstrates comparably decreased mortality rates (5%–19%) and amputation rates (∼2%–12%), which may allude to a lower index of improvement with hirudotherapy compared to its risks (13, 42, 43). These studies primarily cited catheterization injury rather than discussion of sepsis or cardiac disease, and some included procedural intervention such as catheter-directed thrombectomy, so are not representative of our cohort. Acute limb ischemia secondary to ECMO may approach all-cause mortality rates of 27% and amputation rates of ∼18% (44). This may be even higher in SPG (45). Regardless, limb outcome in our surviving patients was dismal in comparison, resulting in amputation or autoamputation in 4/5 leech-treated extremities. One may consider whether prophylactic or earlier leech therapy may be of benefit, however there is not identifiable data to predict onset of acute limb ischemia, and hirudotherapy was generally started soon after recognition of ischemia in survivors (four patients started within two days). The only identified study of prophylactic leech use was for high-risk frostbite in Unani medicine, which limits inference to our cohort (46).

It is also difficult to predict who will develop acute limb ischemia. In one pediatric ECMO cohort, vasopressor score was higher in patients who developed ischemia (28 vs. 13.5), though did not reach statistical significance (44). They also did not demonstrate any predictive statistical significance of commonly proposed risk factors including age, height, cannula site, cannula size (44). Body surface area and cannula size ratio may be a predictor and other studies have demonstrated higher ischemic risk in femoral than neck cannulation (37, 47). Predicting which limbs will require amputation is similarly difficult, though may include coagulopathy and weight loss in infants, and long bone injury, coagulopathy, and neurologic disorder in adolescents (13). In vasopressor-dependent sepsis, amputation risk may increase with higher sequential organ failure assessment scores, INR, lactate, lower platelet count, and culture positive sepsis (15). It is also likely that loss of venous doppler ultrasonography represents irreversible injury (39). There were not enough patients or surviving limbs in our cohort to attempt statistical correlation of leech duration and quantity with outcomes or identify any predictors of mortality. Subjective color improvement with hirudotherapy, which was documented in all patients, was likely indicative of some relief of venous congestion, rather than improved arterial perfusion.

The lack of consistent protocol for leech use over the study period contributed to difficulty interpreting hirudotherapy effect. In reconstructive surgery, protocols vary without consensus on quantity and frequency of application (5, 6, 10, 11). Salvage benefit in adults distinctly improves with at least 4.5 days of treatment in digit reimplantation (5). It was unclear how well or long each leech fed in our patients. It is known that benzodiazepines and narcotics may affect leech feeding, which were part of all subjects' analgosedation regimen. Varying amounts of heparin, papaverine, antithrombin, and nitroglycerine were used prior to determination of refractory ischemia. Other published non-procedural treatments for SPG which were not used in our cohort include aspirin, epoprostenol, recombinant tissue plasminogen activator (rTPA), sympathetic blockade and thrombomodulin (19, 48).

Leveraging anticoagulative properties of leech salivary enzymes locally to limb ischemia is proposed as a plausible mechanism to combat microvascular thrombi but does predispose to bleeding. Leeches typically ingest 5–15 ml of blood per feed, with ongoing bleeding from the bite approaching 10 h (36). Severe bleeding and even death has been reported after use of a single leech (27, 49). It is documented to expect blood transfusions in ∼50% of patients, which may average 3–6 units during hirudotherapy (5, 11). Some report coagulopathy and arterial insufficiency as contraindications to hirudotherapy (6, 9, 49). Prevalence of bleeding adverse events may be 70.2% with ECMO, independent of leeches (50). All but one patient in this study reported discontinuation of leeches due to bleeding, although estimated blood loss varied highly on day of discontinuation (0.26–58.6 ml/kg). Two patients had leech migration and significant bleeding specifically when latching occurred at web space between 1st and 2nd toes. Proposed mechanisms to help isolate leeches and prevent migration include cups, xeroform, and sutures (51–54). These were not documented in our cohort. Despite bleeding, no concerning effect on hemodynamics (VIS or lactate) occurred during the leech phase. Blood transfusion requirement during leech phase increased compared to pre-leech in five patients, albeit with high variability (1.17–8.17 times baseline). The other two patients' decrease in transfusion requirement were explained by either minimal leech use or massive hemorrhage in the pre-leech phase. We were unable to identify any apparent risk factors for leech effect on severity of bleeding or transfusion needs, though ECMO patients generally had higher total bleeding and product transfusion across all phases. There was no consistent improvement in transfusion requirement in the post-leech phase, which was confounded by severity of multiorgan failure at end of life in some patients. Interpretation of transfusion requirements is therefore guarded, though 4/7 patients had 40 ml/kg requirement on at least one day of leech phase (Supplementary Figure S4), which may be considered life-threatening bleeding (55). Lack of general transfusion guidelines results in known provider variance on transfusion decisions (56). Resource consumption of blood products must be weighed against the apparent low beneficial effect of hirudotherapy on limb outcome. Incidentally, 6/7 patients had O blood type, which does not have known association with limb ischemia, however, may have predisposed to worse bleeding (57).

This cohort did not encounter any infection attributed to leech therapy. Transmission of microbes from the leech happens primarily from regurgitation of intestinal contents during feeding, which may be instigated by disrupting a leech's meal (58). Aeromonas species remain the classic microbials of concern, and antibiotic resistance patterns continue in multiple drug classes owing to agricultural antibiotic use and leech farming techniques (58, 59). Prophylactic treatment during leech therapy remains standard in reconstructive surgery, and although there exists no consensus guideline, this would be our recommendation as well. Some advise targeted sampling of leech storage water to identify resistance patterns (60). Given overall decreased use of quinolones (tendinopathies) and trimethoprim/sulfamethoxazole (bone marrow suppression) compared to adults, in PICU populations a 3rd-generation cephalosporin (ceftriaxone) appears most appropriate.

This study has several limitations. The small sample size obviates any formal statistical analysis. It is retrospective and spans a long clinical period that included variable methodology for both limb ischemia treatment and leech use amongst providers. The patient populations have differing and complex diagnoses with additional confounding coagulopathies. Data collection had dependence on both objective and subjective variables in the electronic medical record, including reliability on nursing summary notes, which lacked standardized data elements for recording leech monitoring and outcomes. Interpreting certain data variables associated with leech therapy lack comparable literature to assess their significance (i.e., EBL/kg, TBP/kg) although general definitions of bleeding severity in pediatric critical care are published (55, 56).

## Conclusion

This case series establishes baseline data for use of hirudotherapy in critically ill children with acute limb ischemia caused by arterial malperfusion. Based on this retrospective cohort of critically ill children, we cannot recommend routine use of hirudotherapy for acute limb ischemia from arterial malperfusion in the pediatric intensive care unit. Application of leeches should be aligned with a protocol defining start and stop parameters, standardized leech utilization, and monitoring for adverse outcomes. Future study would benefit from consensus definitions of study outcomes, including perfusion recovery, tissue/limb salvage and bleeding manifestations. Additional prospective studies are needed prior to any standard or systematic recommendations for use.

## Data Availability

The raw data supporting the conclusions of this article will be made available by the authors, without undue reservation.
