# Supplementary figures and images for: Hirudotherapy for limb ischemia in the pediatric intensive care unit: A retrospective observational cohort
Source: Front Pediatr. 2023 Jan 5;10:1011171. doi: 10.3389/fped.2022.1011171 (PMC9849792; doi:10.3389/fped.2022.1011171)

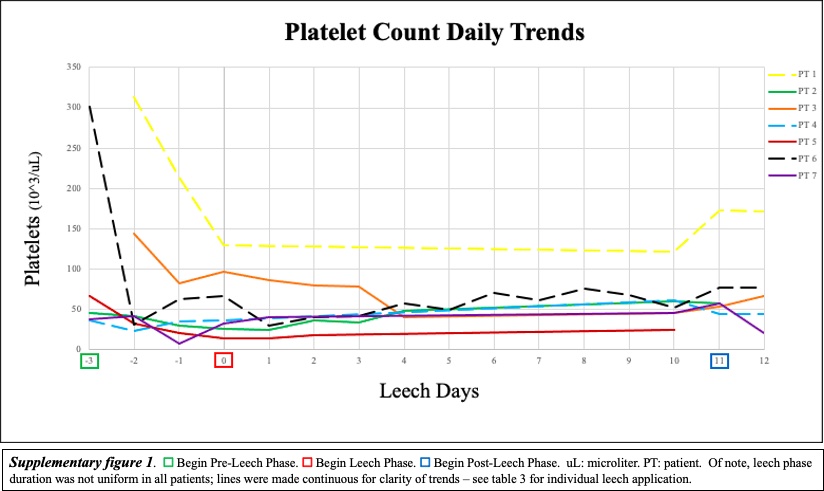

Supplement: Supplementary file 1 [file Image1.jpeg]

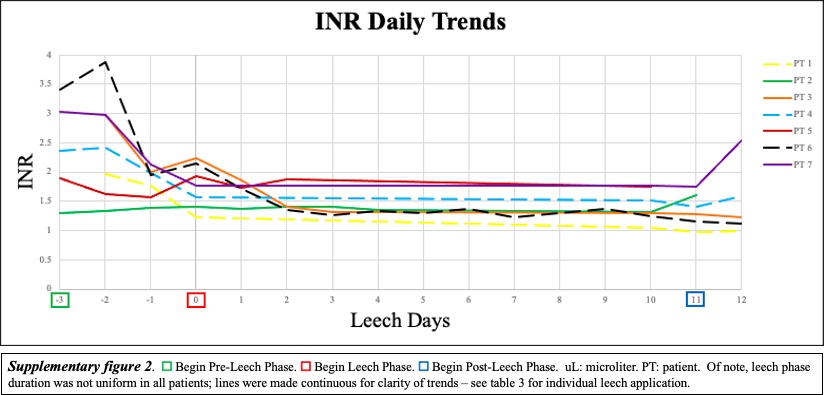

Supplement: Supplementary file 2 [file Image2.jpeg]

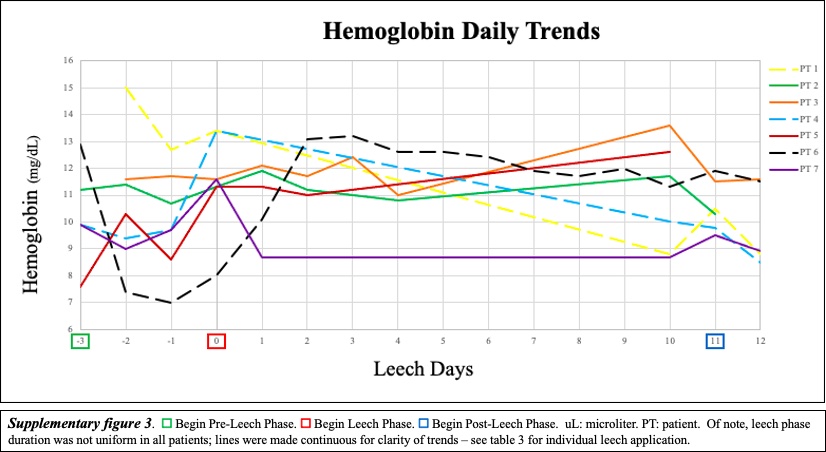

Supplement: Supplementary file 3 [file Image3.jpeg]

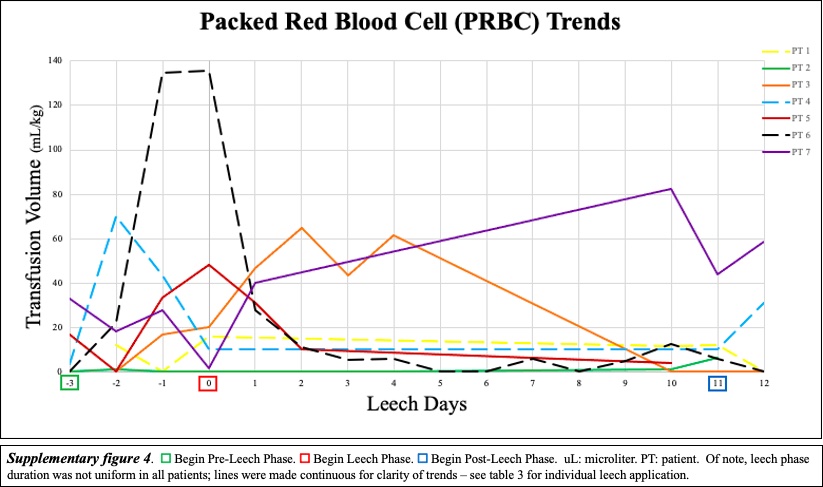

Supplement: Supplementary file 4 [file Image4.jpeg]
